# An LMNB1 Duplication Caused Adult-Onset Autosomal Dominant Leukodystrophy in Chinese Family: Clinical Manifestations, Neuroradiology and Genetic Diagnosis

**DOI:** 10.3389/fnmol.2017.00215

**Published:** 2017-07-18

**Authors:** Yi Dai, Yaling Ma, Shengde Li, Santasree Banerjee, Shengran Liang, Qing Liu, Yinchang Yang, Bin Peng, Liying Cui, Liri Jin

**Affiliations:** ^1^Department of Neurology, Peking Union Medical College Hospital, Chinese Academy of Medical Sciences Beijing, China; ^2^Department of Neurology, Suide Branch Hospital, Yulin First Hospital Yulin, China; ^3^Department of Cell Biology and Medical Genetics, School of Medicine, Zhejiang University Hangzhou, China; ^4^School of Life Science and Biopharmaceutical, Guangdong Pharmaceutical University Guangzhou, China; ^5^Neurosciences Center, Chinese Academy of Medical Sciences Beijing, China

**Keywords:** autosomal dominant adult-onset demyelinating leukodystrophy (ADLD), *LMNB1* gene, multiplex ligand-dependent probe amplification (MLPA), target exome capture, Chinese pedigree

## Abstract

Autosomal dominant adult-onset demyelinating leukodystrophy (ADLD) is a very rare neurological disorder featured with late onset, slowly progressive central nervous system demyelination. Duplication or over expression of the lamin B1 (LMNB1) gene causes ADLD. In this study, we undertook a comprehensive clinical evaluation and genetic detection for a Chinese family with ADLD. The proband is a 52-year old man manifested with autonomic abnormalities, pyramidal tract dysfunction. MRI brain scan identified bilateral symmetric white matter (WM) hyper-intensities in periventricular and semi-oval WM, cerebral peduncles and middle cerebellar peduncles. The proband has a positive autosomal dominant family history with similar clinical manifestations with a trend of genetic anticipation. In order to understand the genetic cause of the disease in this family, target exome capture based next generation sequencing has been done, but no causative variants or possibly pathogenic variants has been identified. However, Multiplex ligand-dependent probe amplification (MLPA) showed whole duplication of *LMNB1* gene which is co-segregated with the disease phenotype in this family. This is the first genetically confirmed *LMNB1* associated ADLD pedigree from China.

## Introduction

Autosomal dominant adult-onset demyelinating leukodystrophy (ADLD) [MIM #169500] is a rare neurological disorder characterized by gradually increasing loss of white matter (WM) within the central nervous system (Coffeen et al., 2000). Majorly, leukodystrophy is manifested with early onset and started appearing in childhood. But the classical clinical symptoms of ADLD, such as autonomic dysfunction, ataxia and cognitive impairment, started to appear in the fourth or fifth decade of life. Brain MRI of ADLD patient’s usually identified with demyelination in brain and spinal cord WM (Schuster et al., 2011). It has previously reported that the familial form of ADLD has associated with chromosome 5q23–31 (Padiath et al., 2006). Padiath et al. (2006) first identified that the heterozygous duplications of the lamin B1 gene (LMNB1, chr5q23.2) is associated with ADLD. Hence, ADLD is related with the duplication or over expression of *LMNB1* gene which leads to produce increased levels of LMNB1 protein. ADLD is one of the rare neurological disorders associated with copy number variation of a candidate gene.

*LMNB1* is a member of the intermediate filament protein superfamily. LMNB1 protein plays a significant structural role by forming the lamina of inner nuclear membrane which maintains the nuclear integrity (Ferrera et al., 2014). LMNB1 protein also plays a regulatory role in gene expression during DNA replication (Tang et al., 2008). Previously, it has been reported that over-expression of LMNB1 protein leads to increase the nuclear rigidity *in vitro* which supports from the nuclei of the skin fibroblast of ADLD patients (Ferrera et al., 2014). Expression of LMNB1 protein is high in oligodendrocytes, which in turn responsible for myelin deposition in the CNS. Overexpression of LMNB1 protein causes demyelination by down-regulating the proteolipid protein through regulating the binding of Yin-Yang 1 transcription factor (Heng et al., 2013).

In our present study, we identified the classical whole duplication in *LMNB1* gene related ADLD in a Chinese family. We also demonstrated the significant usefulness of multiplex ligand-dependent probe amplification (MLPA) in course of clinical diagnosis of genetic disease caused by a copy number variation.

## Materials and Methods

### Subjects

The pedigree under investigation is a Native Han Chinese family from Northern China. Peripheral blood was collected. This study was carried out in accordance with the recommendations of the review board of the Peking Union Medical College Hospital with written informed consent from all subjects. All subjects gave written informed consent in accordance with the Declaration of Helsinki. The protocol was approved by the review board of the Peking Union Medical College Hospital.

### Clinical Evaluation

We performed comprehensive neurological examinations. We explored global cognition by Mini Mental State Examination (MMSE) and Montreal Cognitive Assessment (MoCA). Both results were corrected for age and education according to standardized values referring to the Chinese population. Brain MRI studies were performed using a 1.5 Tesla system. Routine blood test and cerebrospinal fluid test were performed. EEG was investigated.

### Target Exome Capture Based Next Generation Sequencing

DNA samples obtained from the proband were sequenced using target exome-based next-generation sequencing. Roche NimbleGen’s (Madison, Wisconsin, WI, USA) custom Sequence Capture Human Array was used and designed to capture 88,233 kb of targeted sequence, covering 181 exons and flanking sequence (including the 100 bp of introns) of 54 genes (*ABCD1, ACOX1, ALDH3A2, ARSA, ASPA, CSF1R, CYP27A1, DARS2, EARS2, EIF2B1, EIF2B2, EIF2B3, EIF2B4, EIF2B5, FAM126A, FUCA1, GALC, GBE1, GFAP, GJA1, GJC2, HEPACAM, HSD17B4, HTRA1, L2HGDH, MLC1, NOTCH3, PEX1, PEX10, PEX12, PEX13, PEX14, PEX16, PEX19, PEX2, PEX26, PEX3, PEX5, PEX6, PEX7, PLP1, POLR3A, POLR3B, PSAP, RNASEH2A, RNASEH2B, RNASEH2C, SAMHD1, SLC17A5, SOX10, SUMF1, TREM2, TREX1, TYROBP*) which is associated with autosomal dominant cortical infarcted cerebral artery disease with WM lesions and yielded an average of 6,366,534 reads per sample, with approximately 68.78% mapping to the targeted regions. The average sequencing depth of the target area is 204.21 with 98.93% coverage. The procedure for preparation of libraries was consistent with standard operating protocols published previously. In each pooling batch, 10–33 samples were sequenced simultaneously on Illumina HiSeq 2500 Analyzers (Illumina, San Diego, CA, USA) for 90 cycles (specially designed by us for this study). Image analysis, error estimation and base calling were performed using Illumina Pipeline software (version 1.3.4) to generate raw data. The raw reads were screened to generate—clean reads∥ followed by established filtering criteria. Clean reads with a length of 90 bp were aligned to the reference human genome from the NCBI database (Build 37) using the Burrows Wheeler Aligner (BWA) Multi-Vision software package with output files in—bam∥ format. The bamdata were used for reads coverage in the target region and sequencing depth computation, SNP and INDEL calling, and CNV detection. First, a novel three-step computational frame work for CNV was applied. Then, SNPs and INDELs were called using SOAPsnp software and Sam tools pileup software, respectively. A SNP or INDEL was be filtered if it could not follow the criterion: supported by at least 10 reads and >20% of the total reads. The frequency filter was set at 0.05. If a SNP frequency was more than 0.05 in any of the four databases (dbSNP, Hapmap, 1000 Genomes Project, the 124 healthy reference samples sequenced in this study), it would be regarded as a polymorphism, but not a causative mutation.

Last, SNVs were retrieved in The Human Gene Mutation Database^1^ and the Leiden Open Variation Database^2^, and then labeled as reported or novel.

### MLPA Experiment

Follow the instructions for MLPA kit (SALSA probe P071) of MRC-Holland Company in the Netherlands. Probe hybridization, probe linkage, PCR amplification, product capillary electrophoresis and other steps. Finally, the experimental data are processed and analyzed by Genemapper software.

## Results

### History

**Figure 1:**
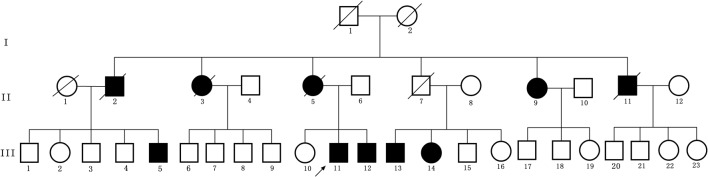
Pedigree structure of the three generation Chinese family with adult-onset demyelinating leukodystrophy (ADLD). Family members with ADLD are indicated with Shading. Squares and circles denoted males and females, respectively. Individuals labeled with a solidus were deceased. Roman numerals indicate generations. Arrow indicates the proband (III-11).

The first symptom of the proband (III-11) is occult-onset constipation around 50 years of age. Then he experienced evident sexual dysfunction. Due to there were several family members suffered from the same disease, he went to our hospital. At that time, he had no obvious motor complaints. Table 1 shows the clinical features in this family. Figure 1 shows the pedigree.

**Table 1:** Comprehensive clinical details of all the family members.

Family ID	Sex	WT/MT	Present age (Years)	Onset age (Years)	Clinical symptoms
I-1	M	—	Died	—	Cause of death are unknown
I-2	F	—	70 (Died)	—	Decreased motor control, bed-ridden for a long time, blindness, then died several years later.
II-2	F	—	75 (Died)	65	Involuntary head movement, decreased motor control, bed-ridden
II-3	M	—	73 (Died)	64	Decreased motor control, bed-ridden.
II-5	F	—	66 (Died)	58	Constipation as onset symptom, then bed-ridden
II-7	M	—	56 (Died)		Die of nasopharyngeal carcinoma, no obvious symptoms when he was alive.
II-9	F	—	69	65	Involuntary head movement, decreased motor control, bed-ridden
II-11	M	—	67 (Died)	56	Constipation as onset symptom, then bed-ridden
III-1	M	—			
III-2	F	—			
III-3	M	—			
III-4	M	—			
III-5	M	—		42	Constipation
III-6	M	—			
III-7	M	—			
III-8	M	—			
III-9	M	—			
III-10	F	WT			
III-11	M	MT	52	50	Constipation
III-12	M	MT		45	Constipation
III-13	M	—		53	Constipation
III-14	F	—		49	Involuntary head movement, Deafness.
III-15	M	—			
III-16	F	—			
III-17	M	—			
III-18	M	—			
III-19	F	—			
III-20	M	—			
III-21	M	—			
III-22	F	—			
III-23	F	—			

### Clinical Evaluation and Tests

The neurological examination of the proband (III-11) identified brisk tendon reflexes, positive Chaddock sign, Hoffmann sign and palmomental reflex. Left ankle clonus was positive. There is NO obvious cerebellar dysfunction.

Global cognitive function measured by MMSE was normal with score of 27. While the MoCA score was 21, which was slightly decreased to the range of Mild Cognitive Impairment (MCI).

The tests of HIV virus, JC virus, thyroid function, arylsulfatase A, galactosylceramidase, α-galactosidase, β-galactosidase, β-hexosaminidase A, hexosaminidase (A + B), very long chain fatty acid (VLCFA) were negative. The tandem mass spectrometry analysis for organic acidemia was negative.

### Neuroradiology

**Figure 2:**
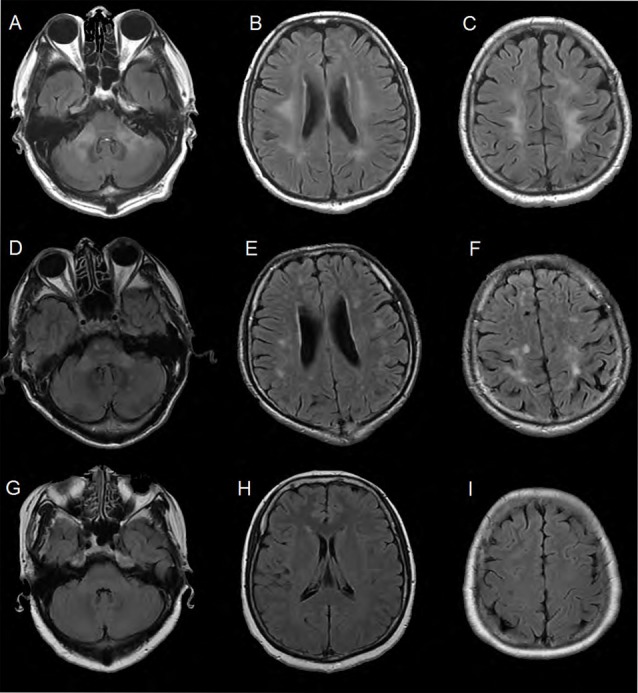
Brain MRI of the family members. **(A–C)** Were FLAIR images of the proband. There were symmetric confluent long T2 signals in mid-cerebellar peduncles, periventricular areas and centrum semi-oval respectively. **(D–F)** Were obtained from the proband’s younger brother (III-12). The images showed patchy hyper-intensities in the same or adjacent planes, which was milder than the proband’s scan. **(G–I)** Were images of the proband’s elder sister (III-10). There were no white matter (WM) lesions in the same areas.

The MRI brain scan of the family members showed that there were symmetric confluent long T2 signals in mid-cerebellar peduncles (Figure 2A), periventricular areas (Figure 2B) and centrum semi-oval (Figure 2C), respectively in the proband (III-11). MRI brain scan of the proband’s younger brother (III-12) showed patchy hyper-intensities in the same or adjacent planes, which was milder than the proband’s scan (Figures 2D–F). MRI brain scan of the proband’s elder sister (III-10) showed no WM lesions in the same areas (Figures 2G–I).

### Target Exome Capture Based Next Generation Sequencing

Next generation sequencing did not identify any causative or pathogenic variants in the proband.

### MLPA

**Figure 3:**
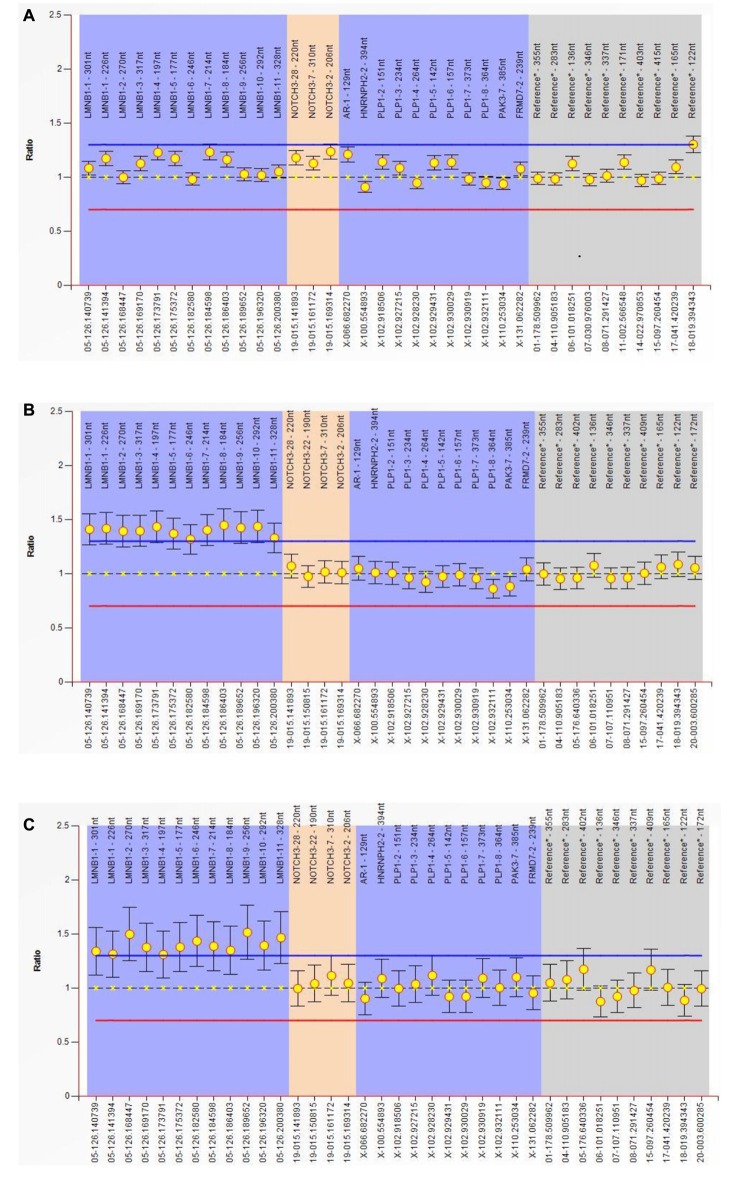
Multiplex ligand-dependent probe amplification (MLPA) experiment. Result show that all of the exon (exon 1–11) of the *LMNB1* gene were repeatedly mutated, the copy number of the entire gene is changed from 2 to 3. The ordinate is the number of copies of the amplified fragments, the abscissa is the chromosomal position coordinates of the amplified fragment. Different color bands play a differentiating role; the same color is identified as the same gene **(A–C)**. MLPA of III-10 **(A)**, III-11 **(B)** and III-12 **(C)** has been described.

MLPA experiment result shows that all of the exon (exon 1–11) of the LMNB1 gene was repeatedly mutated; the copy number of the entire gene was changed from 2 to 3. The ordinate is the number of copies of the amplified fragments; the abscissa is the chromosomal position coordinates of the amplified fragment. Different color bands play a differentiating role; the same color is identified as the same gene (Figures 3A–C).

## Discussion

In our present study, we identified a three generation Chinese family with ADLD. After clinical diagnosis, MLPA found that whole genomic duplication for LMNB1 gene occur in patient and other affected family members of this family. The whole LMNB1 gene duplication is co-segregated with disease phenotype in this family.

Gene duplication through recombination is a spontaneous process having positive force for genome evolution. In addition, duplication of genes also shows dominant negative effect and leads to causes Mendelian disorders. Several affected families with ADLD from different ethnicity, has been reported with duplication of the *LMNB1* gene. Over expression of any gene leads to the presence of excess protein probably occurs in all cells throughout the body. It has been described that a large Italian family with clinical symptoms similar with ADLD and molecular analysis showed that the disease was segregated with the *LMNB1* locus, and over expression of LMNB1 mRNA and LMNB1 protein has been identified among all the affected family members. But comprehensive genetic analysis found that no alteration or copy number variation for *LMNB1* gene (Brussino et al., 2010).

However, Eldridge et al. (1984) reported the first case of ADLD in a large kindred of American-Irish family with a progressive and fatal neurological WM disorder (Eldridge et al., 1984). In addition, later, ADLD has been reported in other population or ethnic groups including Italian, French-Canadian, Japanese, Swedish, and French (Asahara et al., 1996; Marklund et al., 2006; Melberg et al., 2006; Meijer et al., 2008; Brussino et al., 2009, 2010). But the Japanese family reported by Asahara et al. (1996) was only diagnosis by clinical manifestations and the pattern of inheritance, the family didn’t undergo gene detection. In this study, we report the first genetic diagnostic ADLD case in a three generation Chinese family. To our knowledge, this is the first *LMNB1* related pedigree reported in East Asian population.

All the previous reports of ADLD associated with Chr. 5q23–q31 usually showed very similar clinical diagnostic symptoms. Generally, patients presented with autonomic dysfunction as a primary clinical symptom followed by loss of fine motor control or gait disturbances in the fourth or fifth decade of life (Schwankhaus et al., 1994; Coffeen et al., 2000). Various forms of bowel and bladder dysfunction along with sexual dysfunction are reported as the first presenting symptoms (Brussino et al., 2010). Similarly, our patient presented constipation as the first symptom around 50 years old. Constipation is a common symptom of the person suffered from the disease in this family.

However, cerebellar dysfunctions characterized by ataxia, nystagmus, dysmetria and action tremors are also major clinical symptoms for ADLD patients from different ethnic group. On the other hand, pyramidal tract impairment also influences the walking and upper limb function. Despite the proband had not manifested these symptoms, the patients in the first and second generations showed involuntary head movement, impaired movement control and then bed-ridden in the end (see Table 1). All these symptoms are typical manifestations in ADLD. In regard to movement problem, there are clinical variations in the reported family. The Italian kindred reported with ADLD were not presented with Ataxia (Brussino et al., 2010). Meijer et al. (2008) reported in a French-Canadian kindred only manifested as slightly affected dysdiadochokinesis.

Additionally, computed tomography (CT) and MRI scans are very important procedures for diagnosis of ADLD patients. Before neurologist recognized ADLD as a distinguished disease entity, several ADLD patients were clinically diagnosed as primary progressive multiple sclerosis (MS; Eldridge et al., 1984). Now we all know the features of brain MR scan in ADLD is distinctive, similar to other leukodystrophy, which help us to distinguish ADLD from MS (Costello et al., 2009). Most often MRI scan for ADLD patients showed diffuse, confluent, symmetrical fronto–parietal and cerebellar WM abnormalities (Schwankhaus et al., 1994; Coffeen et al., 2000; Melberg et al., 2006; Brussino et al., 2009). Gradually, occipital and temporal lobes are also involved in more advanced stage ADLD patients (Schwankhaus et al., 1994). Melberg et al. (2006) also showed that in Swedish ADLD patients, the WM changes in the posterior part of the atrophied corpus callosum. Involvement of spinal cord in ADLD patients were also identified by MRI scan (Sundblom et al., 2009). Mild atrophy of cerebral and cerebellar hemisphere and medulla oblongata were also reported (Schwankhaus et al., 1994; Melberg et al., 2006).

In this study, we find genetic anticipation in the pedigree. The patients of the second generation typically had the onset around 60 years old, while those of the third generation reported the first manifestation around 50 years old. This pattern is not reported in the previous study. Maybe the development of medicine makes the patients realize their disease earlier.

Regarding the pathophysiological mechanism, there are two major types of lamins in Mammalian cells, namely, A (LMNA) and B (LMN) types. Mutation in any of them leads to a wide range of human diseases, generally called laminopathies (Worman and Bonne, 2007; Worman et al., 2010). Overexpression of B-type lamins leads to growth of nuclear membrane as well as the formation of intranuclear membrane (Prüfert et al., 2004; Ralle et al., 2004). In neural and glial cell, overexpression of *LMNB1* results in increased surface area of nuclear membrane, intranuclear aggregates, perturbations of inner nuclear membrane proteins, chromatin organization, and nuclear pore transport which all together lead to defects in oligodendroglia differentiation (Lin and Fu, 2009). In addition, duplication or overexpression of LMNB1 causes formation of abnormal intranuclear membranes which finally results in altered subcellular localization of nuclear envelope proteins and perturbed nuclear transport.

In conclusion, we describe the first Chinese ADLD family in which disease pathogenesis is caused by the “classical” molecular mechanism of LMNB1 duplication, which expand the geographic and ethnic map of this disease.

## Author Contributions

YD, YM, SLi, SB and LJ designed the study. SLiang performed the molecular diagnosis by Sanger sequencing. SB carried on the bioinformatic analysis. YD and QL contributed to the molecular diagnosis analysis based on NGS. YM, BP, LC, YY and LJ worked on the clinical study. YD and SLi contributed to the Sanger sequencing validation and MLPA. YD, SLi and LJ wrote the article. All authors read and approved the final manuscript.

## Conflict of Interest Statement

The authors declare that the research was conducted in the absence of any commercial or financial relationships that could be construed as a potential conflict of interest.

## Footnotes

^1^http://www.hgmd.cf.ac.uk/ac/index.php

^2^http://www.lovd.nl/3.0/home
